# Efficient known ncRNA search including pseudoknots

**DOI:** 10.1186/1471-2105-14-S2-S25

**Published:** 2013-01-21

**Authors:** Cheng Yuan, Yanni Sun

**Affiliations:** 1 Department of Computer Science and Engineering Michigan State University East Lansing, MI, 48824, USA

## Abstract

**Background:**

Searching for members of characterized ncRNA families containing pseudoknots is an important component of genome-scale ncRNA annotation. However, the state-of-the-art known ncRNA search is based on context-free grammar (CFG), which cannot effectively model pseudoknots. Thus, existing CFG-based ncRNA identification tools usually ignore pseudoknots during search. As a result, dozens of sequences that do not contain the native pseudoknots are reported by these tools. When pseudoknot structures are vital to the functions of the ncRNAs, these sequences may not be true members.

**Results:**

In this work, we design a pseudoknot search tool using multiple simple sub-structures, which are derived from knot-free and bifurcation-free structural motifs in the underlying family. We test our tool on a contiguous 22-Mb region of the Maize Genome. The experimental results show that our work competes favorably with other pseudoknot search methods.

**Conclusions:**

Our sub-structure based tool can conduct genome-scale pseudoknot-containing ncRNA search effectively and efficiently. It provides a complementary pseudoknot search tool to Infernal. The source codes are available at http://www.cse.msu.edu/~chengy/knotsearch.

## Background

**Figure 1:**
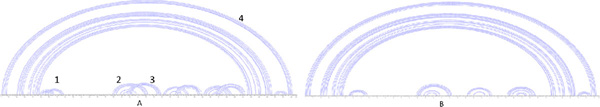
**Consensus secondary structure of tmRNA and the secondary structure described by SCFG (pseudoknots missing)**. A. Consensus secondary structure of RF00023 (tmRNA) in Rfam. Stacking base pairs in 1 are parallel to base pairs in 2 and 3. 1, 2, and 3 are nested in 4. 2 and 3 form a pseudoknot. B. Secondary structure described by SCFG (pseudoknots missing).

Noncoding RNAs (ncRNAs), which function directly as RNAs without translating into proteins, play diverse and important biological functions [1]. Many types of ncRNAs function through both their sequences and secondary structures, which are defined by interactions between Watson-Crick and wobble base pairs. Pseudoknot is a functionally important structural motif in ncRNA secondary structures. In pseudoknots, bases in loop regions can form base pairs with bases outside the stem loop. In a graphical representation where arcs connect base pairs, pseudoknot-free secondary structures only contain parallel or nested base pairs while pseudoknot structures allow "crossing" base pairs, shown by an example in Figure 1.A.

It is already known that pseudoknots play important functions in telomerase RNA, tmRNA, rRNA, some riboswitches, some protein-biding RNAs, Viral ribosomal frameshifting signals, etc [2]. Different research groups [3,4] have shown that the pseudoknot structure in the telomerase RNA is essential for telomerase activity. Gilley and Blackburn [3] experimentally demonstrated that disruptions of the pseudoknot base pairing within the telomerase RNA from *Tetrahymena thermophila *prevent the stable assembly in vivo of an active telomerase. They further concluded that the pseudoknot topology rather than sequence is critical for an active telomerase. Similarly, biologists reported that the pseudoknots in tmRNA are highly important for protein biding, tmRNA maturation, and proper folding of the tRNA-like domain [5]. Currently, 26,704 sequences in 71 ncRNA seed families of Rfam 10.0 [6] contain pseudoknots. With the advances of sequencing technologies and structure predictions, more pseudoknot structures are expected to be revealed.

Because the functions of ncRNAs are determined by both the sequence and structure, successful ncRNA homology search tools must consider both sequence and structural conservations. Existing ncRNA search tools can be divided into two categories. One is commonly referred to "known ncRNA search", which aims to detecting homologs of ncRNAs with annotated secondary structures. The second category includes tools for identifying novel ncRNA genes. This work belongs to the first category and focuses on ncRNAs containing pseudoknots.

For pseudoknot free ncRNAs, the state-of-the-art search method is based on stochastic context-free grammars (SCFGs), which can accurately model the evolutionary changes of both the sequences and structures of a group of homologous ncRNAs. Commonly used general and specialized known ncRNA search tools such as Infernal [7], RSEARCH [8], and tRNAScan-SE [9] are all based on SCFG. In conjunction with the ncRNA family database Rfam, Infernal has been successfully applied to classify query sequences into different types of ncRNA. However, SCFGs are not able to model pseudoknot. Thus, the implementations of SCFG by Infernal neglect pseudoknots in the structures. For example, although RF00023 (tmRNA) has four pseudoknots, its SCFG only models the knot-free structures, shown in Figure 1.B. As a result, Infernal could misclassify sequences as members of families containing pseudoknots. In addition, Infernal has high computational cost, limiting its usage in large-scale data sets, such as those generated by the next-generation sequencing technologies.

More complicated grammars such as context-sensitive Grammars (CSGs) [10] exist to faithfully model pseudoknots. However, the computational cost of the parsing algorithms of a CSG is even higher than using a CFG. Besides CSGs, other grammars such as parallel communicating grammar systems [11], RNA pseudoknot grammars [12], tree adjoining grammars (TAGs) [13,14], and multiple context-free grammars [15] have been proposed to model pseudoknot structures. These work described the grammars and associated parsing algorithms. However, they have not been widely used in pseudoknot search in large-scale databases. First, although the parsing algorithms are polynomial, their cubic or even higher time or memory complexity [15] limits their large-scale applications. Second, these methods were designed for and tested on secondary structure derivation rather than homology search. In order to conduct large-scale homology search, local parsing algorithms are needed. As there are no source codes or executable implementations of these grammars, it is not clear whether they can be automatically applied to known ncRNA search including pseudoknots.

In this work, we design an efficient pseudoknot search algorithm for all types of pseudoknots. Our method is based on a set of carefully chosen *simple sub-structures *(or *sub-structures *for short), which do not contain pseudoknots or bifurcations. The time complexity of the parsing and probability computation algorithms for an SCFG including the CYK, the inside, and the outside algorithm will be significantly reduced when the secondary structure does not contain any bifurcation [10,16]. Thus, these simple sub-structures can be searched efficiently using existing implementations of SCFGs. For multiple sub-structures extracted from one ncRNA family, we choose a set of sub-structures according to their sizes and false positive (FP) rates in order to maximize the search performance. These chosen sub-structures will be used in a progressive search. Our experimental results show that our tool competes favorably with other pseudoknot search methods.

## Related work

Brown and Wilson [17] proposed an RNA pseudoknot search method using intersections of SCFGs. Both Brown's method and our approach try to decompose pseudoknot into knot-free structures for SCFG modeling. There are two major differences. First, our sub-structures are not only knot-free, but also bifurcation free, which enables faster search. Second, while Brown and Wilson's method focused on the model construction and parsing algorithm, we focus on choosing an optimal set of sub-structures to optimize the search performance. The model construction and the parsing algorithms can be conveniently implemented using Infernal, which has gone through extensive testing.

Structural motifs similar to sub-structures have been used as filters to speed up Infernal. FastR [18] relies on stem-loops ((*k*, *w*)-stack) that do not contain bulge or interior loops to search for ncRNAs. Weinberg et al. [19] use more flexible structural motifs based on sub-CMs and profile HMMs for ncRNA classification. Smith [16] used a decision tree to organize partial SCFG models for fast ncRNA search. Currently, these filters are only designed and tested for speeding up SCFG search.

Available pseudoknot search tools include RNAv [20] and RNATOPS [21]. RNATOPS designs a graph model for RNA pseudoknots and solves the structure sequence alignment by graph optimization. RNAv is a profile based RNA secondary structure variation search program that detects distant ncRNA structural homologs, which might be missed by RNATOPS.

The chain filter designed by Zhang et al. [22] consists of a collection of short conserved words in an ncRNA family. In our work, we use a collection of simple sub-structures for pseudoknot search. Similar to Zhang et al.'s work, we find that using a collection of simple structures can achieve a good tradeoff between sensitivity and false positive rate during search.

## Approach

There are two components in the method. The first component is the design of a set of sub-structures to represent an ncRNA family. The second component is a progressive search strategy using the designed sub-structures. Different regions of an ncRNA sequence have different sequence and structural conservations. Well-conserved structural and sequence motifs tend to yield better search performance than poorly conserved motifs. Our approach sorts sub-structures extracted from different regions according to their lengths and predicted FP rates in order to choose a set of sub-structures with the optimal search performance.

**Figure 2:**
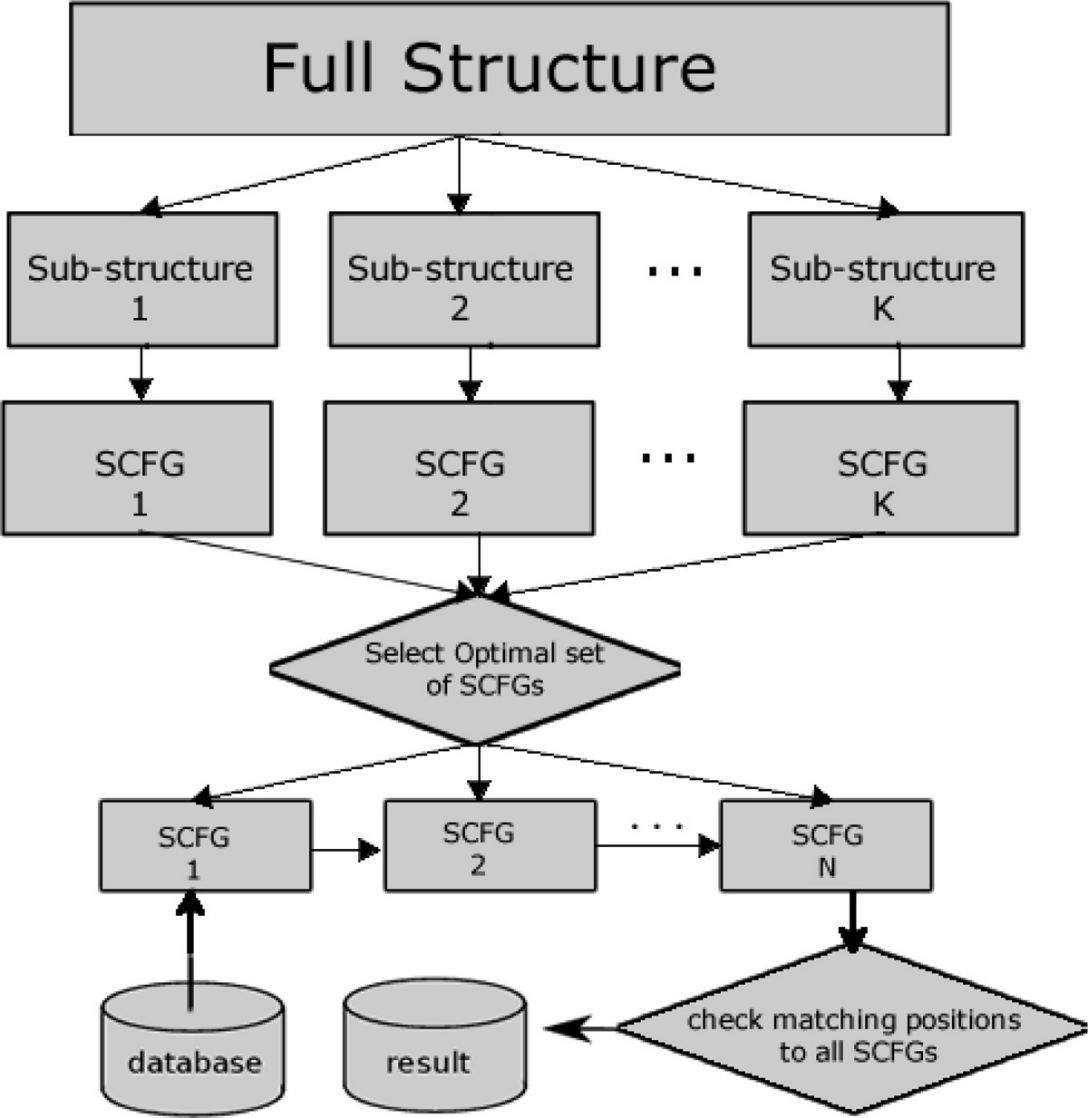
**The pipeline of the SCFG construction and the progressive search**.

For a chosen set of sub-structures, we conduct a progressive search according to a pre-determined order. During the progressive search, one sub-structure is only applied to regions containing matches to all previous sub-structures. A sequence is classified into the pseudoknot family if and only if 1) it passes the score thresholds of all the chosen sub-structures; 2) the position relationship between matched substrings is consistent with the relationship between the sub-structures. Thus the false positive rate of the chosen set of sub-structures is bounded by the product of the false positive rates of all component sub-structures. The pipeline of the approach is illustrated in Figure 2.

### Sub-structure derivation

**Figure 3:**
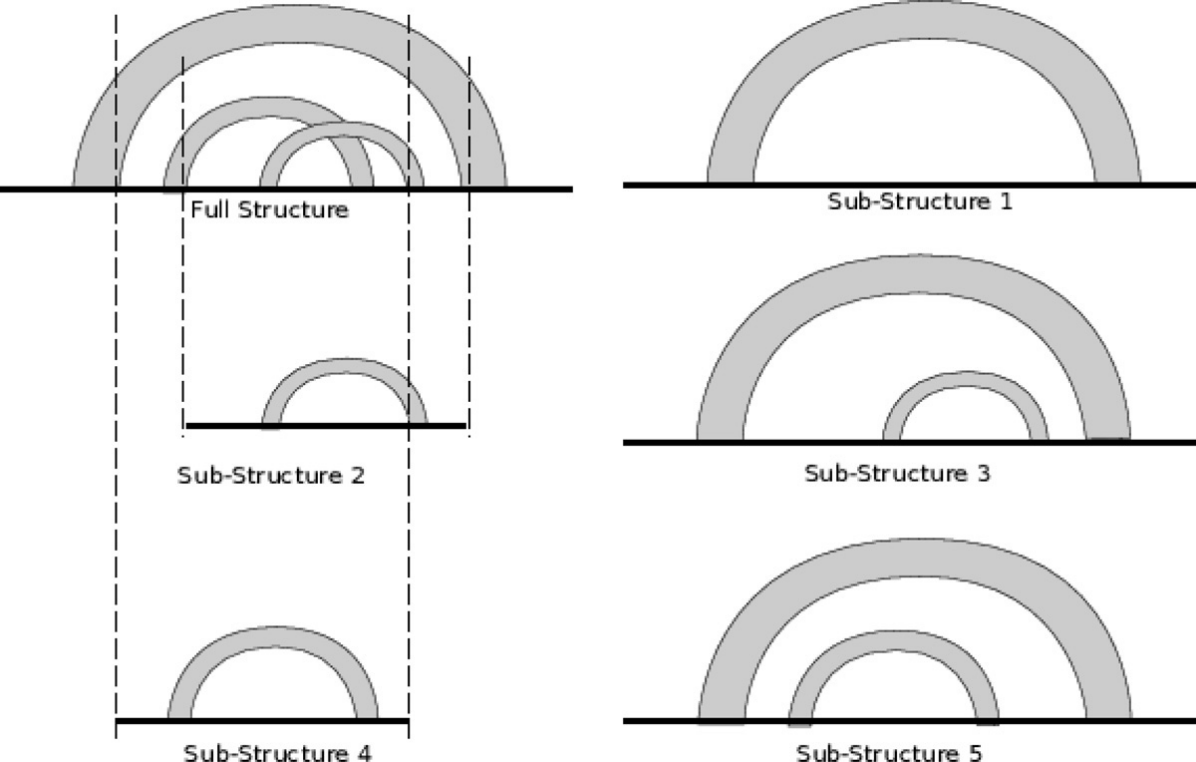
**Five candidate sub-structures can be constructed from three stems in a pseudoknot structure**. Each arc represents a stem containing nested base pairs and possible internal/bulge loops. Single-stranded regions are represented using solid lines.

In order to use SCFG-based models for pseudoknot search, we decompose a pseudoknot structure into simple sub-structures. Each sub-structure contains at least one *stem*, which includes a set of stacking base pairs allowing short bulge and interior loops. A full secondary structure of an ncRNA family can be decomposed into multiple stems. Combinations of stems define different sub-structures. Figure 3 shows all five simple sub-structures derived from the given pseudoknot.

We describe a method to systematically extract all simple sub-structures from a pseudoknot. In the first step, all stems are extracted and sorted in increasing order of their starting positions (i.e. 5' end of the outmost base pair in the stem). Second, we build a bit table R of size N by N for N stems extracted from the first step. For each cell *R*[*i*, *j*], if stem *i *and stem *j *are nested, *R*[*i*, *j*] = 1; otherwise, *R*[*i*, *j*] = 0. Table R provides us information about whether given stems can form one sub-structure. Given the stem set and their relationship table R, we use pseudocode in **Algorithm 1 **to extract all simple sub-structures. In the pseudocode, *H^x ^*is the set of sub-structures containing *x *stems. Thus, the union of *H^x ^*for *x *= 1 to *N *consists of all simple sub-structures for a given secondary structure. The number of sub-structures depends on the number of nested stems. Suppose the average number of nested stems inside a stem is *n*. The total number of sub-structures is *O*(*N *+ *N*2^*n*^).

**Algorithm 1 **ExtractSubstructures **Input: **a secondary structure containing pseudoknots **Output: **all simple sub-structures

1:   **for **each stem *i *= 1 to *N ***do**

2:      /* h: a sub-structure containing a set of stems */

3:      h = {*i*}

4:      *H*^1 ^= *H*^1 ^∪{*h*}

5:   **end for**

6:   **for ***L *= 2 to *N ***do**

7:      *H^L ^*= Ø

8:      **for **each sub-structure *h *∈ *H*^*L*-1 ^**do**

9:         **for **each stem *i *∉ *h ***do**

10:            /* h[i] is the ith stem in a sub-structure h */

11:            **if **R[h[1], i] and R[h[2], i] ... and R[h[L-1], i] **then**

12:            /* construct a new sub-structure *h*' */

13:            *h*' = *h*∪{*i*}

14:            *H^L ^*= *H^L ^*∪{*h*'}

15:            **end if**

16:         **end for**

17:      **end for**

18:   **end for**

19:   output all sub-structures *H *= *H*^1 ^∪*H*^2 ^∪ ... ∪*H^N^*

Algorithm 1 only outputs the combination of stems. For each stem (or stem set) in a sub-structure, we add loop and flanking regions using the following three rules. Let the 5' and 3' ends of the outmost base pair in a sub-structure be *I*_5 _and *I*_3_, respectively. Thus, *I*_5 _<*I*_3_.

• Rule 1: Add all single-stranded regions including bulge and internal loops between *I*_5 _and *I*_3_.

• Rule 2: Except the base pairs inside the chosen stems in a sub-structure, all other base pairs will be treated as single-stranded regions.

• Rule 3: Extend the flanking single-stranded regions to the left of *I*_5 _and to the right of *I*_3 _until the first base pair in other sub-structures.

### Search performance of different sub-structures

**Figure 4:**
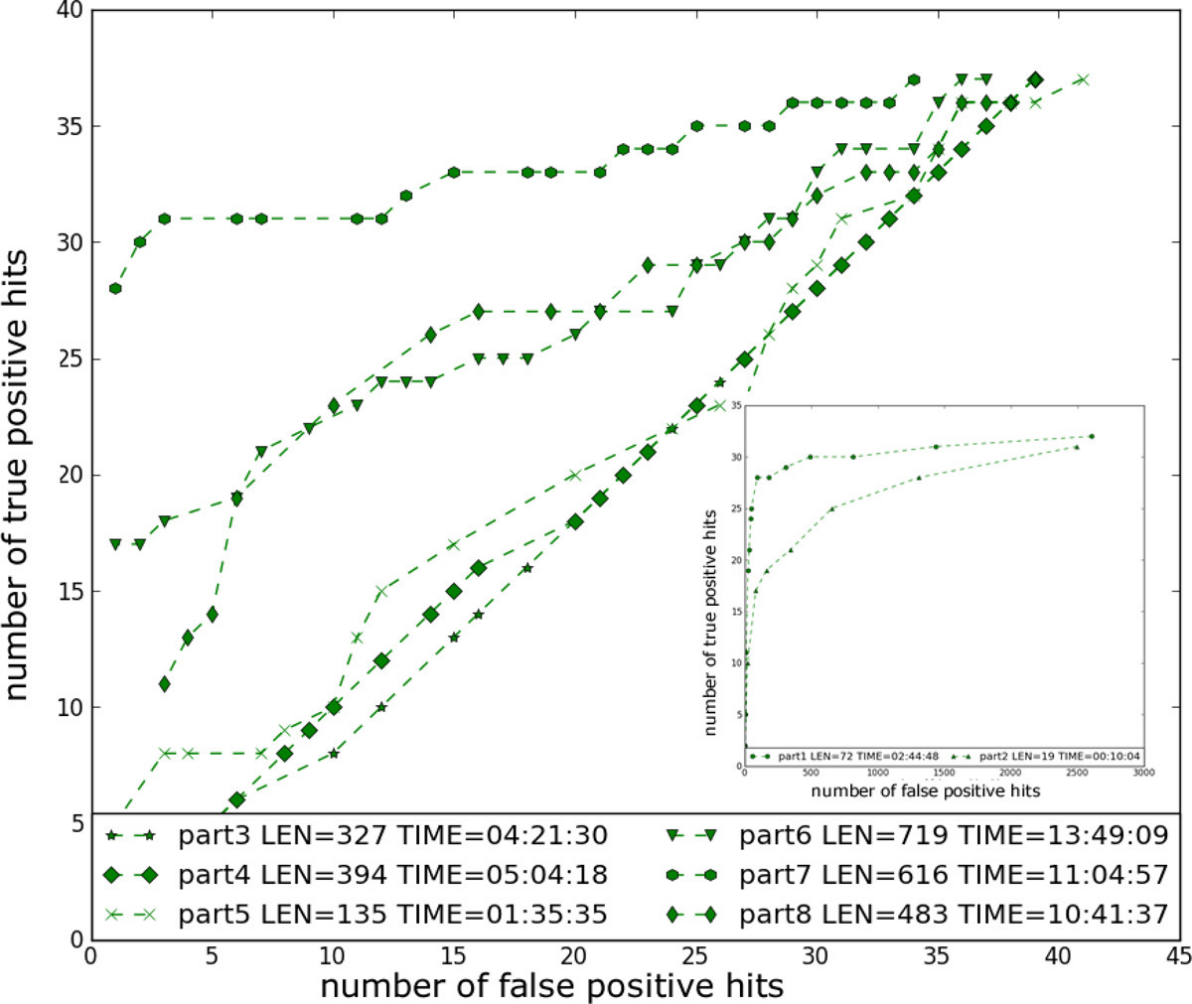
**Number of TP hits and FP matches of each sub-structure under different score thresholds**. For each sub-structure, the length and the search time corresponding to the highest sensitivity is listed. Time format is hr:min:sec. Due to highly different number of FP hits, two sub-structures are plotted in the embedded figure.

Each sub-structure can be conveniently modeled by an SCFG. As different sub-structures are derived from regions with different sequence and structural conservations, their corresponding SCFGs have different performance in database search. In this section, we use an example to illustrate this. We built SCFGs for eight sub-structures derived from RF00373 (Ribonuclease P) and evaluated the sensitivity, FP rates, and running time of the eight SCFGs when applying them to a to a 22.5 M Maize genome (data is described in "Experimental results"). The sensitivity and FP rates of different sub-structures from the same family can be compared using true positive (TP) hits and FP hits respectively, because the condition positive and condition negative sets are the same for all sub-structures derived from the same family. For any SCFG $M_{i}$, let the set of matched sequences be $Hit\left(M_{i}\right)$. Let the set of true pseudoknot sequences be *S*, which are the sequences in seed families containing pseudoknots in Rfam. The number of true positive and FP matches of a sub-SCFG is $\left|Hit\left(M_{i}\right)\cap S\right|$ and $\left|Hit\left(M_{i}\right)\backslash S\right|$, respectively. We summarized the TP hits and FP matches of eight SCFGs under different score thresholds in Figure 4. In addition, the search times are included for the score thresholds corresponding to the highest sensitivity. It is clear that different SCFGs have highly search performance. During a progressive search using a series of sub-structures, the number of matches of the preceding sub-structure determines the search space of the current sub-structure. Thus, the total search time depends on both the FP hits and the model running time, which is heavily affected by the model length. In order to maximize the search efficiency, it is important to sort all candidate sub-structures according to their FP rates. When the FP rates of two or more sub-structures are similar (same order), we prefer shorter models because they incur less search times.

#### Sort sub-structures according to their E-values

There are two methods to calculate the FP rates of sub-structures. Theoretically, by assuming a background model for random sequences and applying the CYK algorithm [10], we can directly calculate the probability that a random sequence matches an SCFG model. Empirically, we can apply the SCFGs to a large annotated sequence database and record the number of FP matches. However, as it is more important to compare the FP rates of different sub-structures than knowing their exact values, it is not necessary to directly calculate FP rates. By assuming that the SCFG alignment scores for random sequences follow an exponential distribution, as implemented by Infernal, we can use E-values of the designed score cutoffs to sort all sub-structures.

For an alignment score and a database size, an E-value indicates how many random hits a user can expect to see with the same or better score in a random sequence database of similar size. Thus, E-value indicates FP hits when it can be computed accurately. Currently, we are using the E-value calculation method provided by Infernal. Although the assumed score distribution is not accurate, we found that the estimated E-values allow us to compare FP rates of different sub-structures with high accuracy. In order to estimate E-value, Infernal generates a set of N random sequences whose GC content depends on the covariance model. These N random sequences then are aligned against the model. In this process, all searching result with score > 0 will be considered as hits. Scores of the top *X *hits are assumed to follow an exponential distribution with two parameters, *μ *and *λ*. The maximum likelihood approach is then taken to fit scores of hits into an exponential distribution.

$$E=db*e^{-\lambda \left(score-\mu \right)}$$

where *db *is adjusted database size and is defined as

$$db=\frac{db_{target}}{dbsize_{random}}\left(randhit+0.5\right).$$

In the E-value computation, *μ *and *λ *are parameters trained in Infernal. *sc *is the score for which one needs to calculate E-value. *db_target _*is the size of target database. *db_random _*is the number of random sequences generated for curve fitting. At last, *randhit *is the number of random sequences found by the covariance model. We can directly obtain *μ *and *λ *from each calibrated covariance model, which is built for a sub-structure. With these two parameters available, we can use the above equation to compute E-values for given scores.

Our experiments show that although the change of E-values does not scale with the change of the FP rates, the order of E-values is highly consistent to the order of FP rates for all 71 families we tested. Only for SCFGs with similarly small FP rates, their E-values cannot accurately reflect their order. Table 1 presents an example. It is worth noting that we also considered to use the average entropy to sort the sub-structures. However, our experiments show that there is no systematic relationship between entropy-based measurements and the FP rates of sub-structures.

**Table 1:** The order of E-values is highly consistent to the order of number of the FP hits.

sub-structure	E-value	FP hits	sub-structure	E-value	FP hits
RF00373_part2	1.71e+03	4894	RF00373_part1	7.33e+02	2606
RF00373_part5	7.30e-02	41	RF00373_part3	3.58e-02	39
RF00373_part4	3.18e-06	39	RF00373_part6	5.29e-09	37
RF00373_part8	4.52e-09	39	RF00373_part7	3.40e-15	34

#### Choose sub-structures for progressive search

During a progressive search based on multiple sub-structures, the final sensitivity is bounded by the lowest sensitivity of all sub-structures. The final search time and FP rates heavily depend on the order of applying these sub-structures. Let the final array of sub-structures be $SUB=\left(H_{1},...,H_{i},...,H_{n}\right)$, where $H_{i}$ will be applied before $H_{j}$ if *i *<*j*. Let the size of the original database be L. For a sub-structure $H_{i}$, let *t_i _*and *fp_i _*be its search time per hit and FP rate, respectively. The final FP rate is bounded by $\overset{n}{\underset{i=1}{\prod }}fp_{i}$. The final search time is roughly $T=L\overset{n}{\underset{i=1}{\sum }}t_{i}\left(\overset{i-1}{\underset{j=1}{\prod }}fp_{j}\right)$, where $L\overset{i-1}{\underset{j=1}{\prod }}fp_{j}$ is roughly the search space for the sub-structure $H_{i}$. Minimizing T requires the accurate computation of *t_i _*or quantification of the relationship between *t_i _*and *fp_i_*, which is not known as a priori. Although Infernal provides estimated running time, it can be quite different from the true running time. According to the equations, it is clear that we should apply short sub-structures with small FP rates before long sub-structures with high FP rates. Thus we develop a greedy algorithm to generate a set of sub-structures for progressive search based on our empirical observations.

We split sub-structures into *short *group and *long *group, which contain short and long sub-structures respectively. For each group of sub-structures, we sort the sub-structures according to their E-values and apply a greedy algorithm to choose a set of sub-structures for search. The main steps of the greedy algorithm are outlined below, starting from the short group:

1. In each iteration, choose the sub-structure with the smallest E-value. Remove it and append it to the final sub-structure list SUB.

2. Remove any remaining sub-structure in both groups that only contains stems in this sub-structure.

3. Repeat the first step until all stems are covered by one chosen sub-structure or the E-values of all remaining sub-structures are bigger than a pre-determined cutoff (default is 1).

If SUB has not included all stems, we apply the same process to the long group and append the chosen sub-structures to SUB. We require all stems covered by the chosen sub-structures in order to ensure the representation of the annotated pseudoknot structure. It is possible that this constraint will exclude homologous ncRNAs that lack annotated stem loop structures. Currently, we use size 150 as the threshold to divide sub-structures into the short and the long group.

### Implementation

For each sub-structure, we train an SCFG-based model based on the corresponding alignment in the training data using Infernal. Let the SCFGs trained from *n *sub-structures of an ncRNA family $SUB=\left(H_{1},...,H_{i},...,H_{n}\right)$ be $\Pi =\left(M_{1},...,M_{i},...,M_{n}\right)$, where $M_{i}$ represents a single SCFG. A sequence can be classified into the corresponding family if the following conditions are satisfied. First, the sequence contains matches to all designed SCFGs in Π. SCFG match will be defined in the following text. Second, for every pair of strings that match two SCFGs, their position relationship must be consistent with the annotated relationship between two SCFGs in the underlying ncRNA family. There are three types of position relationship between two sub-structures: parallel, nested, and cross-over. Cross-over indicates existence of pseudoknots.

We determine SCFG match using score thresholds. For all sequences in the training set, its alignment score with a given SCFG is computed. The minimum score of all the seed sequences is used as the score threshold. This score cutoff is similar to the NC (trusted cutoff) bit score thresholds used in HMMER [23] or Infernal. When the training data contains a good representation of the family member sequences, the computed score threshold can ensure a high sensitivity during homology search. If the training set only contains close homologs of this ncRNA family, the designed cutoff may be too high for remotely related homologs.

## Experimental results

In order to test the performance of our tool for pseudoknot search in sequence databases, we conducted two experiments. First, we examined the automatically classified pseudoknot sequences in Rfam. Second, we applied it to part of the Maize genome. On the same data set, we compared our tool with RNAv, RNATOPS, and Infernal.

### Pseudoknot sequences in Rfam

Because CFG cannot model pseudoknots, the implementations of Stochastic CFG (SCFG), covariance models (CMs) in Rfam neglect pseudoknots in the structures. As a result, tools that use SCFG for ncRNA search such as Infernal could misclassify sequences as members of pseudoknot families. Each Rfam family contains a seed sequence set and a full sequence set. While the seed sequence set contains manually validated homologous sequences, the full sets are automatically produced using SCFG-based search against RFAMSEQ database [6]. Thus, some of the sequences in the full set may not contain pseudoknot structures that are annotated in the seed sequences. We examined the full member set of the 71 ncRNA families containing pseudoknots in Rfam using our tool. Many families contain dozens of sequences that lack the annotated pseudoknot structures. For all those sequences that cannot be matched by our tool, we also utilized the Infernal alignments and a RNA stem finding tool RNAmotif [24] to double check whether the base pairs in pseudoknot structures are missing. The SCFG alignments output by Infernal contains annotations of all base pairs that do not form pseudoknots. By comparing the annotated base pairs and the consensus secondary structure of the seed alignments, we can extract the regions that should form pseudoknots. Then, we applied RNAmotif to output all stems of size at least two in the chosen regions. Failing to output any stems validated our findings that these sequences do not have the annotated pseudoknots. The results are summarized in Table 2. Although homologous ncRNAs may not share the same set of stems, simply ignoring pseudoknots without knowing their impacts on the function can introduce a large number of false members. In particular, it was already experimentally shown that pseudoknot structures are vital to the functions of some types of ncRNAs [3-5]. For these well-studied pseudoknot structures, it is important to include them during homology search.

**Table 2:** Sequences that do not contain annotated pseudoknots and thus may not be real members.

ID	seqs without knots/num of seqs	ID	seqs without knots/num of seqs	ID	seqs without knots/num of seqs	ID	seqs without knots/num of seqs
RF00009	37/500	RF00010	3/3864	RF00011	26/460	RF00023	53/2871
RF00024	56/233	RF00028	2587/39045	RF00030	47/470	RF00041	2/151
RF00140	81/524	RF00176	37/64	RF00216	25/126	RF00233	22/76
RF00259	78/124	RF00261	43/78	RF00499	1/16	RF00523	2/5177
RF00622	1/94	RF01050	3/60	RF01072	21/271	RF01073	1/7006
RF01086	15/1093	RF01087	1/31	RF01089	4/25	RF01096	2/45

### Data set preparation

We created a simulated data set based on a contiguous 22-Mb region of the Maize Genome [25]. The annotation of the 22-Mb region does not contain any hit to the 71 pseudoknot families in Rfam. In order to evaluate the sensitivity of pseudoknot search tools, we randomly chose 1,586 out of 26,704 seed sequences from 71 pseudoknot families and inserted them in the 22-Mb region. The remaining seed sequences are used as the training data. In order to examine the FP rate of SCFG-based tools, we also created 1,586 sequences without pseudoknots. Specifically, for each of the 1,586 seed sequences, we altered the bases to disrupt the base pairs that can form pseudoknots. Similarly RNAmotif is applied again to ensure these sequences lose the annotated pseudoknot structure. These modified 1,586 sequences and the original 22-Mb region of the Maize Genome constitute the negative training data. Any hit to them is an FP hit. Note that by changing the bases, the modified sequences might share lower sequence similarity to the trained model and thus pose an easier case for all tools. Even so, our experimental results still show that different tools exhibit highly difference performance on this data set. Thus, we feel this data set is a reasonable test set.

There are two major advantages of using this simulated data set for testing pseudoknot search tools. First, as the 22-Mb region of the Maize genome does not harbor any reported ncRNA that contains pseudoknots, we can measure the empirical FP rates of pseudoknot search tools with higher reliability than using simulated sequences, which are usually generated using a simple i.i.d. model or low-order Markov model. In particular, the Maize genome contains a high percentage of repeats and low-complexity regions, which could not be simply simulated and can pose a challenge for ncRNA search as warned by the Rfam website (http://rfam.sanger.ac.uk/). Second, using thousands of seed members of the pseudoknot families provides us adequate test data for evaluating the sensitivity.

Besides using the seed sequences of Rfam, we also considered another pseudoknot sequence database Pseudobase [26]. This database contains 304 RNA sequences with pseudoknot structures. A majority of them are sub-strings of Rfam seed sequences. Thus, we choose to use Rfam seed sequences as the true label.

### Results and comparisons

In order to separate the training set and the test set, we removed the sequences that were inserted in the Maize genome from the seed alignments. For the alignments composed of the remaining sequences, we trained the full covariance model and the models for the sub-structures. We used the designed sub-structure sets for pseudoknot search. We evaluated the performance of pseudoknot search tools using three metrics: the sensitivity, FP hits, and running time. For each ncRNA family represented by an SCFG M , let Hit(M) be the set of output sequences by a search tool. Let *S *be the set of true pseudoknot sequences, which, in this data set, includes seed sequences of each pseudoknot family. The sensitivity is thus defined as:

$$sensitivity=\frac{\left|Hit\left(M\right)\cap S\right|}{\left|S\right|}$$

Any output that does not overlap with true pseudoknot sequences is a false positive hit. The number of FP hits of a search tool on one family is computed as:

FPhits=|Hit(M)\S|

We report the FP hits instead of the FP rates for two reasons. First, the condition negative set is family specific and thus is the same for all search tools for a given family. Second, the size of the condition negative set is mainly determined by the size of the genome minus the size of all true pseudoknot sequences. For a large genomic sequence, the FP rate becomes very small and cannot reflect the difference between different tools.

On the same dataset, we run RNAv, RNATOPS, and Infernal 1.0.2. Of the three, RNAv and RNATOPS are designed for pseudoknot search. For Infernal and sub-structure, no hidden Markov model-based filtration was used in order to maximize the sensitivity. Other parameters were set as default for Infernal. We used the default parameters to run RNAv and RNATOPS. All experiments were run on the main cluster of the High Performance Computing Center on campus (http://www.icer.msu.edu/?q=hpcc). Each experiment was allocated four CPU days at most. There are 65 families and 31 families that failed RNAv and RNATOPS, respectively. The search jobs for those families were killed by the cluster after four CPU days. No results were produced. Thus we could not report the results for those families. RNAPTOPS output results for 22 families by the end of the allocated time.

The performance of these four tools is recorded in Table 3. The results show that our tool is significantly faster than RNATOPS and RNAv. For a majority of families, the running time is smaller than half an hour. A closer examination reveals that 99% of the running time is attributed to the first sub-structure, which is expected. Of the six families for which RNAv successfully generated outputs, they all have the sensitivity of 1.0, equal to the sensitivity of sub-structure based search. Of the 40 families for which RNATOPS reported results, 14 of them have equal sensitivity to ours. 1 family yields slightly better sensitivity than ours while other 24 families have significantly worse sensitivity. Thus, overall, our search achieves higher sensitivity than RNAv and RNATOPS. In addition, sub-structure based search tool incurs lower FP rate than RNATOPS and RNAv. Table 3 shows that RNATOPS yields low FP hits. Of the 40 families, RNATOPS has the same number of FP hits as ours for only one family and significantly more FP hits for the rest. In particular, RNATOPS outputs over 1,000 hits for 9 families.

**Table 3:** Sensitivity, FP hits, and running time comparison between RNAv, RNATOPS, Infernal, and sub-structure.

RNA fam ID	sen	FP	time RNAv	sen	FP	time RNA-TOPS	sen	FP	time Sub-structure	sen	FP hits	time INFER-NAL
RF00009							**1.0**	**5**	**01:47:37**	**1.0**	38	26:16:07
RF00010							0.58	**95**	**00:18:47**	**0.97**	318	17:54:31
RF00011							0.84	**25**	**00:06:51**	**0.97**	179	09:09:52
RF00023							0.4	**1**	**00:06:54**	**1.0**	180	13:40:31
RF00024							**0.95**	**24**	**00:06:33**	0.81	86	20:36:42
RF00028							**0.83**	**6**	**22:30:56**	0.72	37	79:05:16
RF00030							0.38	**26**	**02:35:01**	**0.98**	87	83:37:31
RF00041							0.95	**0**	**00:10:37**	**1.0**	64	01:27:52
RF00094							0.88	**0**	**00:09:21**	**1.0**	35	00:54:20
RF00140							0.97	**0**	**01:05:08**	**1.0**	33	01:52:09
RF00165				0.21	4	4 days	**1.0**	**0**	**00:22:10**	**1.0**	14	00:32:25
RF00176	**1.0**	58077	19:54:40				**1.0**	**0**	**00:05:48**	**1.0**	21	00:50:54
RF00216							0.87	**0**	**00:03:03**	**1.0**	30	04:42:29
RF00233				0.26	0	4 days	0.96	**0**	**00:09:06**	**1.0**	29	00:47:38
RF00259							**1.0**	**0**	**00:05:41**	**1.0**	5	02:09:52
RF00261							**1.0**	**0**	**00:13:53**	**1.0**	20	02:50:11
RF00373							0.92	**27**	**01:35:35**	**0.95**	363	14:15:43
RF00381				0.38	30	4 days	**1.0**	**0**	**00:17:10**	**1.0**	15	00:33:42
RF00390				**1.0**	763	4 days	**1.0**	**0**	**00:05:21**	**1.0**	6	00:07:35
RF00458							**1.0**	**0**	**00:09:37**	**1.0**	10	02:18:47
RF00499							**1.0**	**0**	**00:09:51**	**1.0**	115	01:33:43
RF00505				0.2	2	4 days	**1.0**	**0**	00:32:27	**1.0**	5	**00:29:55**
RF00507				0.41	7	4 days	0.95	**0**	**00:34:44**	**1.0**	23	00:52:44
RF00523				0.29	160	4 days	0.95	**24**	00:20:31	**1.0**	145	**00:19:24**
RF00622							**1.0**	**0**	**00:05:15**	**1.0**	14	00:42:40
RF01050							**1.0**	**0**	**00:41:32**	**1.0**	13	39:22:21
RF01072				0.52	273	4 days	0.96	**0**	**00:08:37**	**1.0**	30	00:10:13
RF01073	**1.0**	196631	13:13:32	0.11	3	4 days	**1.0**	**0**	**00:18:36**	**1.0**	13	00:29:04
RF01074				0.5	91	4 days	**1.0**	**0**	**00:06:59**	**1.0**	10	00:15:00
RF01075							**1.0**	**0**	**00:07:59**	**1.0**	7	01:00:46
RF01076	**1.0**	139249	16:20:29				**1.0**	**0**	**00:20:36**	**1.0**	5	00:35:33
RF01077							**1.0**	**0**	00:53:32	**1.0**	4	**00:37:23**
RF01078							**1.0**	**0**	**00:12:38**	**1.0**	3	00:26:04
RF01079				**1.0**	333	4 days	**1.0**	**0**	**00:07:37**	**1.0**	3	00:16:01
RF01080				0.5	135	4 days	**1.0**	**0**	**00:08:04**	**1.0**	110	00:13:41
RF01081				0.67	284	4 days	**1.0**	**0**	**00:06:47**	**1.0**	3	00:08:44
RF01082				0.5	2934	4 days	**1.0**	**0**	**00:05:47**	**1.0**	4	00:09:13
RF01083				**1.0**	3002	4 days	0.67	**1**	**00:04:34**	**1.0**	7	00:07:05
RF01084							**1.0**	**0**	**00:10:46**	**1.0**	8	01:53:25
RF01086							**1.0**	**11**	**05:18:38**	**1.0**	13	05:39:23
RF01087				0.5	3	4 days	**1.0**	**0**	**01:19:41**	**1.0**	12	01:37:01
RF01088							**1.0**	**0**	00:39:09	**1.0**	4	**00:37:14**
RF01089				0.33	1	4 days	**1.0**	**3**	**01:03:09**	**1.0**	20	01:21:27
RF01090				0.43	4	4 days	**1.0**	**0**	**00:23:11**	**1.0**	8	00:36:35
RF01091							**1.0**	**0**	**00:13:06**	**1.0**	4	00:28:51
RF01092	**1.0**	165990	10:58:02	**1.0**	**0**	4 days	**1.0**	**0**	**00:17:57**	**1.0**	15	00:30:08
RF01093				0.42	67	4 days	**1.0**	**0**	**00:13:59**	**1.0**	23	00:29:56
RF01094							**1.0**	**0**	**00:52:46**	**1.0**	3	01:10:47
RF01095							**1.0**	**0**	**00:10:56**	**1.0**	2	00:27:12
RF01096	**1.0**	166314	16:44:20	0.5	1	4 days	**1.0**	**0**	**00:23:04**	**1.0**	2	00:24:45
RF01097				0.25	1	4 days	**1.0**	**0**	**00:12:18**	**1.0**	4	00:22:09

Bold font is applied to the highest sensitivity, the lowest FP hit, or the shortest running time for each RNA family. The empty cells indicate that the corresponding tools did not generate any output within 4 CPU days.

**Figure 5:**
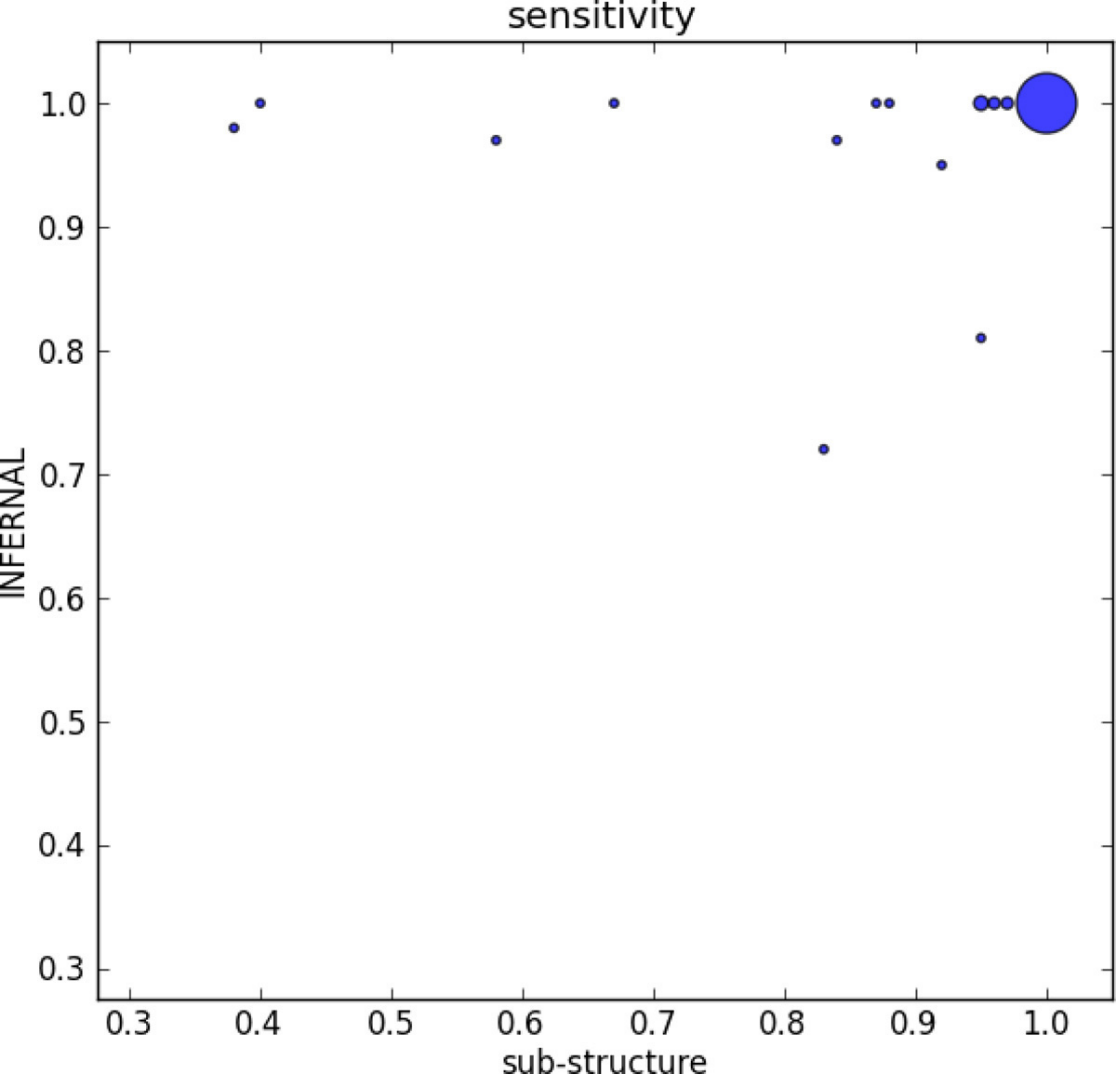
**Sensitivity comparison on 71 families**.

**Figure 6:**
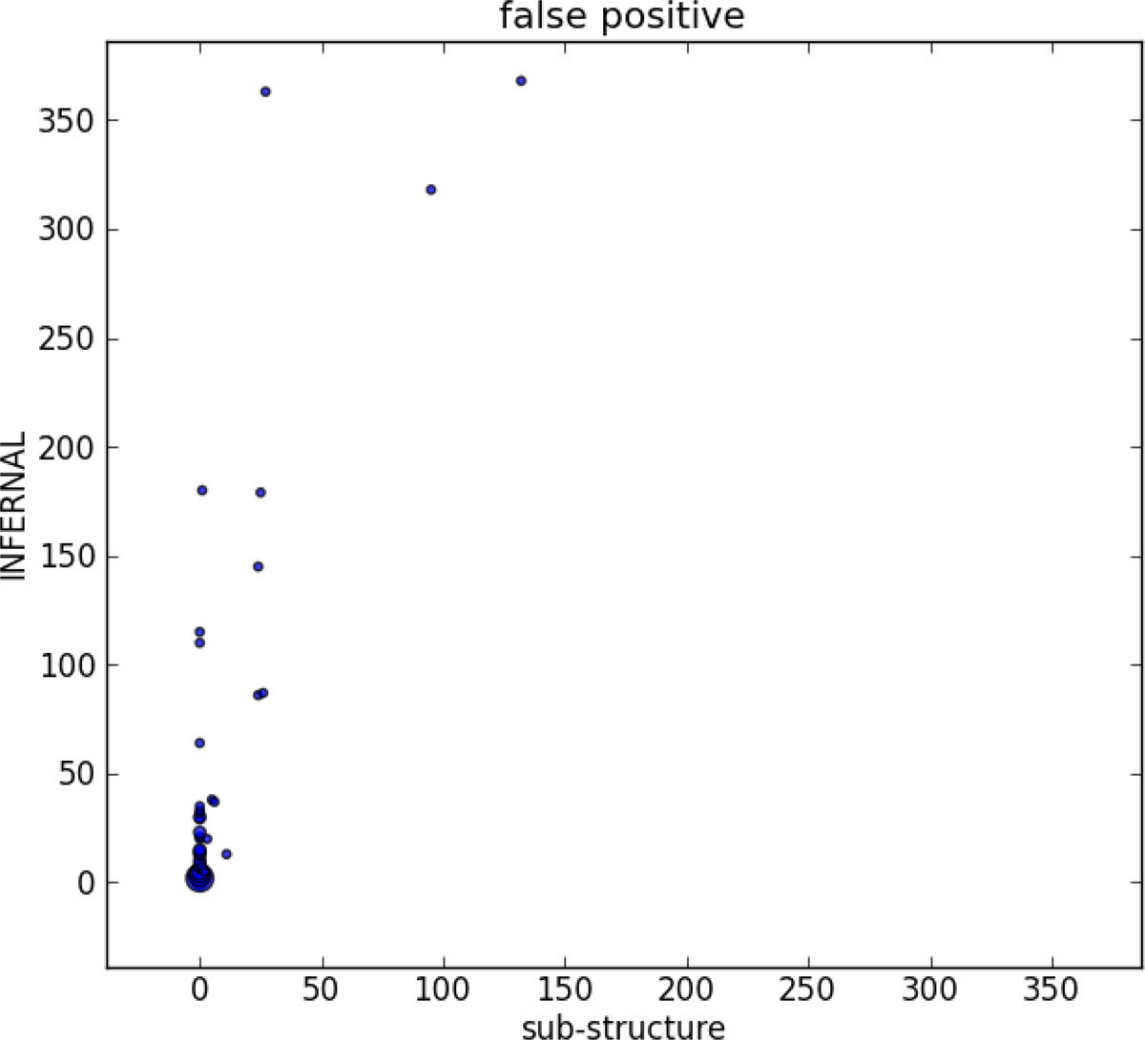
**Comparison of false positive hits on 71 families**.

**Figure 7:**
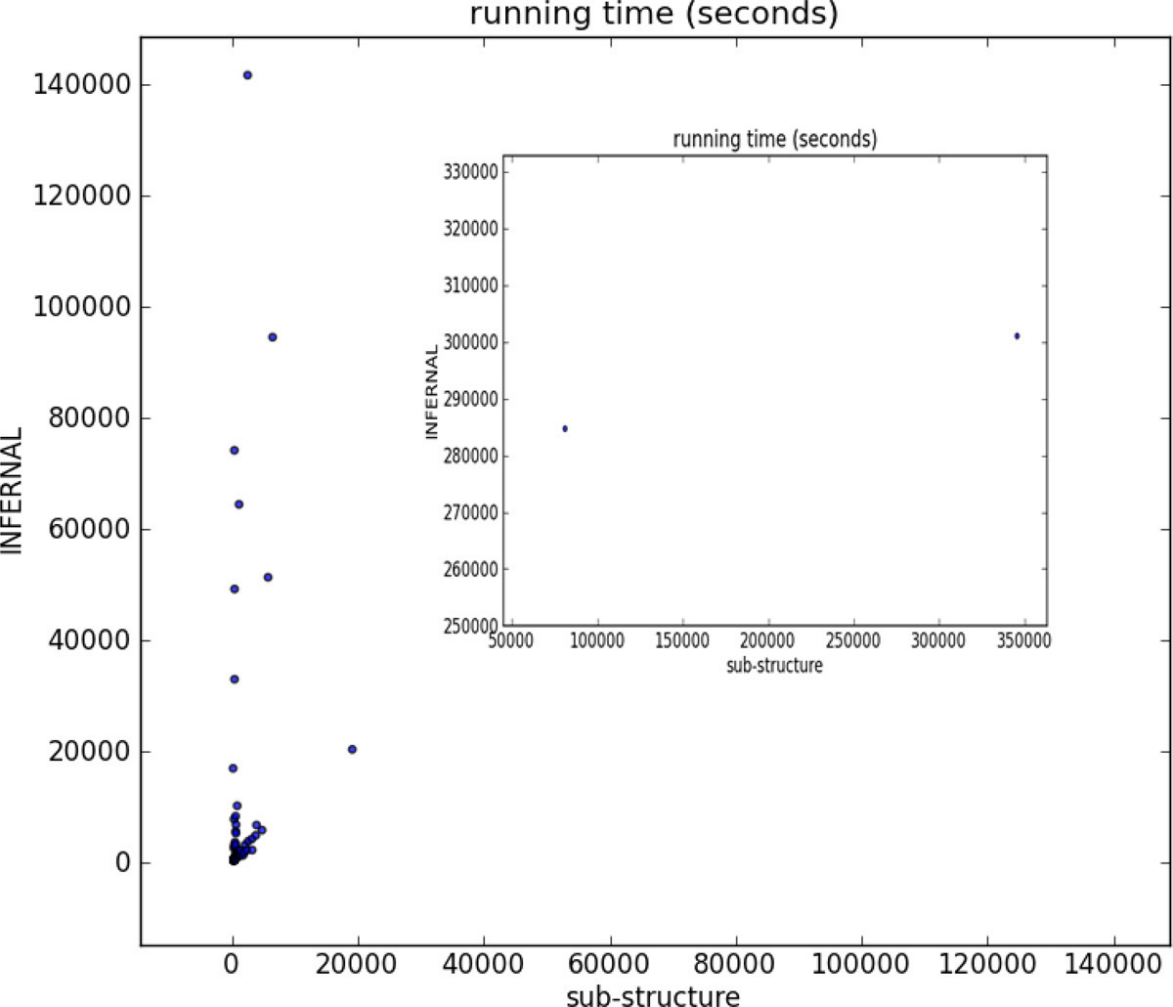
**Running Time comparison**. There are 4 families on which Infernal run much longer than on other families. To keep an appropriate scale, there running times are not displayed on the figure.

We compared the sensitivity, FP hits, and running time of Infernal and our tool in Figure 5, Figure 6, and Figure 7 using X-Y scatter plots. As Infernal and our tool generate the same sensitivity or other metrics for some families, we use the bubble plot to visualize the number of the same data points. As expected, Infernal is highly sensitive. However, it reported dozens of hits on the pseudoknot-free sequences which we inserted as false positive sequences. For all families, Infernal reported equal or more FP hits than our tool. In addition, it is generally slower than sub-structure-based tool. Out of 71 RNA families, sub-structure-based tool has shorter running time on 66 families. For 14 families, it yields 10x speed up over Infernal.

There is no significant difference in the sensitivity between Infernal and sub-structure-based tool when the average sequence length in a family is not too long. Infernal has better sensitivity on longer and more complicated RNA families including RF00010, RF00011, RF00023, and RF00030. The major reason behind our worse sensitivity on the long families is that we use sub-structure that cover every stem. Thus, we only classify sequences that have all characterized stems from the underlying structure. However, some remote homologs may lose base pairs in stems during evolution. Thus while we guarantee to find sequences that have the same structures as the annotated pseudoknots, we can miss some homologs, leading to lower sensitivity for some families.

## Conclusion

Although Infernal is highly sensitive in known ncRNA search, caution must be taken when applying Infernal to ncRNA families containing pseudoknots. In this work, we designed a pseudoknot search method based on a set of carefully chosen sub-structures. These sub-structures do not contain pseudoknots or bifurcations. SCFGs can be conveniently built on them and searched with high efficiency. In order to minimize the overall FP rate and the running time, we sorted sub-structures according to their lengths and their E-values for designed trusted cutoff (NC) bit score thresholds. We designed a greedy algorithm to choose a set of sub-structures and applied the progressive search to minimize search time. Our experimental results showed that our tool competes favorably with RNAv and RNATOPs, both of which have been used for pseudoknot search in large databases. This work provides a complementary pseudoknot search tool to existing SCFG-based knot-free ncRNA search methods.

Currently our tool only reports homologous ncRNAs with the same number of characterized stems as the training data. As a result, some true homologs that have lost one or multiple stems will be ignored. As part of the future work, we plan to incorporate available RNA-seq data for remote homology search.

## Competing interests

The authors declare that they have no competing interests.

## Authors' contributions

YS proposed the original idea and algorithms. YS and CY both contibuted to experiment design. CY conducted the experiments and implemented the algorithms. Both Authors read and approved the final manuscript.

## Declarations

The publication costs for this article were funded by NSF DBI-0953738 and IOS-1126998.

This article has been published as part of *BMC Bioinformatics *Volume 14 Supplement 2, 2013: Selected articles from the Eleventh Asia Pacific Bioinformatics Conference (APBC 2013): Bioinformatics. The full contents of the supplement are available online at http://www.biomedcentral.com/bmcbioinformatics/supplements/14/S2.
